# Mediating effects between social capital and health care utilization in Italy–a structural equation model analysis

**DOI:** 10.1186/s12963-025-00441-6

**Published:** 2025-12-20

**Authors:** Tallys Feldens, Chiara Seghieri, Andrea Fontana, Paolo Berta

**Affiliations:** 1 Istituto di Management, L′EMbeDS, Scuola Superiore Sant ′Anna di Pisa, Piazza Martiri della Libertà, 33, Pisa, Italy; 2 Università degli Studi di Milano-Bicocca, Milan, Italy

**Keywords:** Social capital, Healthcare utilization, Italy

## Abstract

**Background:**

Social capital, in its broad definitions, has been linked to improved health outcomes, yet the scarce consistency of social capital measurements and its further effects on healthcare utilization remain less clear. Particularly in Italy, where regional disparities and an aging population challenge the healthcare system, understanding these dynamics is crucial. This study proposes two population-based indicators of social capital and investigates whether they influence health itself and healthcare utilization.

**Method:**

Italian population data from 2014 to 2023 was used to develop two social capital measurements: Social support and Social participation, applying Item Response Theory (IRT). Hence, we applied structural equation modeling (SEM) to explore the pathways between social capital, self-reported health status, and healthcare utilization. The analysis includes control variables for demographic and behavioral factors.

**Results:**

Our main findings contribute with the current literature by identifying that population-based measures for social support and social participation may be useful for empirical research, and both direct and indirect effects of social constructs were found significantly associated with health and health utilization outcomes. Both social participation and social support were found to exert significant positive effects on self-perceived health and health utilization. The model suggests that while better social connections contribute to improved health, such increased support and participation can also lead to increased healthcare-seeking behavior.

**Conclusion:**

Social capital plays a dual role in shaping both health outcomes and healthcare utilization in Italy. Our findings highlight the relevance of social resources as population-level determinants of health and access, suggesting that strengthening community networks and health literacy can reduce inequities and enhance the efficiency of healthcare systems.

**Supplementary Information:**

The online version contains supplementary material available at 10.1186/s12963-025-00441-6.

## Background

### Motivation

In Italy, health care systems are still being reshaped after the COVID-19 pandemic – and the reliance on a decentralized health care system by region weights into significant heterogeneity on access and utilization of health care services. Parallelly, the country still struggles with the regional differences in distribution of health care professionals and quality of delivered care [1, 2]. Environmental variables, lifestyle factors and the important role of social and familiar nets contribute expressively to the elevated longevity in Italy, which ends up adding more pressure into the health care system [3].

In addition to the classic health determinants (physical environment, socio-economic conditions, genetics, and others), recent literature has been consistently pointing to an additional determinant of health: social capital [4]. Several works argue in favor of beneficial impacts of social support, social participation and other dimensions of social connection on health outcomes [5–10]. Still, only a few studies have dedicated to understanding whether these effects are followed by a decrease in health utilization or not [11, 12].

Apart from the possibility of affecting health utilization, it is yet to be clarified whether social aspects of the individuals can affect health utilization directly, or if such effect is exclusively mediated by the effect on health outcomes [6, 8, 12]. Besides, it is important to understand whether these effects remain important after controlling for observable demographic and behavioral factors.

Current literature shows competing streams of thought pointing to different transmission channels of such social capital effects. There are those who sustain social capital has a protective effect by improving well-being and promoting preventive behaviors [5, 6, 8, 12–16], and those who argue in favor of a pushing effect towards health access once someone is in need [5, 13, 14, 17–20]. As there is enough empirical evidence for both arguments, we take advantage of causal modelling to test these specific pathways altogether.

One of the challenges of estimating social capital effects is the lack of a single population-based measurement for “social capital” that allows a direct comparison with health outcomes and health care utilization data. For instance, one study [5] evidencing the impact of social capital on Italian’s health has chosen the frequency of meeting with friends as a proxy of structural social capital. However, by evaluating one single aspect of the social life of an individual, one risks overseeing the multidimensionality of social connections.

Our study aims to contribute to this literature by proposing two population-based measurements using Item Response Theory: one for social support, and another for social participation. Then, we try to acknowledge direct and indirect impacts of social capital via such two different perspectives. Our estimation strategy assumes mediating effects and controls for several possible confounders, using the framework of structural equation modelling for the Italian population between the years of 2014 to 2023.

### Theoretical and empirical evidence

Social capital has been discussed by sociologists from the 1980s. Since then, many have used the term to define it as the social resources that derive from an embedment and social connections [21]. It can be categorized between formal (or institutional) or informal. Particularly, formal social capital is represented by participation in organizations and activities such as political, educational, recreational and others alike. In the other hand, informal social capital is represented by the circle of family, friendships, neighbors, and others [21]. As the first theories vary in definitions and did not specifically indicate a single measurement to the so-called social capital, this term has been used to proxy multidimensional elements of the social aspect of life such as group participation, trust in institutions, trust in others, support from friends and relatives, professional networks and many others [21]. However, all these proxies probably represent a manifestation of social capital instead of social capital [22]. Nonetheless its multiple definitions found on applied work, social dimensions of life are empirically linked to health outcomes due to the relevant role it has on people’s wellbeing [6–11, 15, 19].

Theoretical explanations of how social capital may impact health propose three plausible pathways: (i) by psychosocial processes, (ii) by health behaviors and (iii) by access to services and amenities [21, 23]. The psychosocial pathway stems from the well-established association between loneliness and mental health, which stresses the linkage between scarce or deficient social connections with the occurrence of anxiety and depression [24–26]. One branch of the psychosocial pathway relies on the buffering argument. Here, social support is often thought of as a protective factor against other determinants of health and distressful moments in life [5–7, 13, 16, 21]. The literature suggests that the sense of community, belonging, purpose in life, and self-enhancement derived from social participation and support are feelings connected to lower cognitive impairment and lower mortality [16, 25, 26].

The second pathway arguments that having strong social networks or social support can enhance healthy lifestyles, help to adhere to health treatments and promote prevention [5, 6, 13–15].

On their turn, the third pathway focus on the access to health services. Studies that used such focus sustain that people with more intense social connectedness are more prone to demand health care [13, 17]–[18]. Besides, people can seek advice from their close friends and relatives to decide visiting emergency department once they are suffering from an acute condition [18, 27]. Turnbull et al. [18] explains that the social network helps to validate the sense-making of the symptoms, the urgency of the need, and decreases the burden of deciding whether to access health care and how. Besides, social capital can act as an informal referral system where people can get informed about healthcare procedures, health insurances and health professionals, thus improving health literacy and influencing the seek for health care utilization [5, 13, 14, 17–20, 28]. As social capital appears to influence at the same time health itself, health behaviors and health care utilization, it is needed to disentangle these possible effects on a pathway analysis that allows to capture each connection independently. Due to the scarce literature in this regard, we are especially interested in understanding the effects on health and healthcare utilization. Still, there are only a few studies that acknowledge the mediation characteristic inherent of this relationship. In particular, as there are studies pointing to benefits in health outcomes and others indicating that social support and participation may influence the seek for help, the net effect on healthcare utilization remains unclear.

For example, Demirer et al. [6] on trying to estimate effects for social support on mental health, had only acknowledged the chance of social support being affected by the multimorbidity and then affecting mental health; but not possible direct effects of social support on health or on healthcare utilization. Wang et al. [8], in its study on health behaviours of post stroke patients, had modelled a pathway analysis characterizing that social support could influence patient’s health behaviors, but not health itself, or health system’s utilization. Lai and Ma [9] modelled social support as a mediator between mental health struggles and risky behaviors but had not consider possible direct health outcomes stemming from social support. Noh and Park [7] considered the connection between social support and mental health, but only as a mediator, not as a possible direct factor.

Luo et al. [10] was one of the few studies that considered the potential direct impact of social support on health, while also considered two indirect channels, one through perceived stress and another through self-efficacy. They found that social support influences stress and self-efficacy and exerts a direct effect on subjective well-being. Still, their approach has not focused on how these effects could be translated into health care utilization. On the other hand, Harber-Aschan et al. [11] have developed a model where social networks are found to influence unplanned care accesses, however, only directly and not through health status.

Another limitation of the current literature is that only a few other studies have adjusted for relevant sociodemographic variables. For instance, Luo et al. [10] have controlled their pathway analysis by gender, age, education and location; Wang et al. [8] by gender, age, education and income; Noh and Park [7] have only used gender and economic status; Mo et al. [15] instead stratified by age alone, while Yang et al. [29] did not mention controlling covariates at all.

It is of particular interest knowing whether formal and informal social capital behave similarly or differently in such setting. Building upon these insights, our model, described in the following section, aims to address the gaps identified in the literature by examining the direct and indirect effects of social capital on health outcomes and health care utilization, thereby providing a more comprehensive understanding of these interconnected pathways.

## Methods

### Data

We used data from the annual cross-section multi-scope survey “Aspects of Daily Life” from the Italian National Institute of Statistics (ISTAT). Years between 2014 and 2023 were selected to maximize data availability^1^. We elected adult individuals from 18 years old, from all Italian regions, whose representativeness was inherited from the design of the survey.

In order to develop two population-based measurements of social capital based on social capital theories, we built two indicators, one for social participation, representing the “formal” social capital and another for social support, thus representing the “informal” one. In our estimation, we define social participation as a score that represents group and social activities one is taking part in; and social support is another score that represents the quality and quantity of close relationships such as family, friends and neighbors. We theorize that although both dimensions of social capital may enhance health outcomes, they show slightly different interactions with the environment: while social support intends to capture intimate and close relationships, social participation is meant to cover the sense of belonging to a community and the interaction of the individual with the society. Besides, we add flexibility to the model to explore the direction, magnitude and relative importance of these two components of social capital in our estimates. While for social support we constructed an index using quality and quantity of the relationships, for social participation we used the grouping defined by Durante et al. [22].

The items from the ISTAT survey we used to build the two scores are depicted in Table 1 below. Rather than averaging the measures [11] or selecting a single measure to play the proxy of the estimation [5], we decided to use a data-driven method to select the level of social capital that can be assessed in base of the respondent’s answers.

The two scores were calculated using Item Response Theory (IRT) from Samejima (1997) [30], where each score considers the graded characteristic of the alternatives given to the respondents and creates a parameter that represents the latent trait we are theoretically implying – one representing social support and the other representing social participation. IRT estimates were developed using the R package “mirt” [31]. Further information about the IRT indexes is available on the Supplementary material.

**Table 1:** Item composition of social capital scores – social support and social participation

Social support	Social participation
AMICI2 – Do you have friends with whom you can count to?	FINAS – In the last 12 months have you given money to an association?
PARENT – Do you have relatives with whom you can count to, except your parents, children, siblings, grandparents and grandchildren?	VOLON - In the last 12 months have you participated in a non-paid activity for voluntary association?
VICINI – Do you have neighbors with whom you can count to?	ATGRA - In the last 12 months have you participated in a non-paid activity for non-voluntary association?
RELAM – What is your level of satisfaction with the relationship with your friends in the last 12 months?	PGRVO - In the last 12 months have you attended meetings of voluntary association?
RELFAM - What is your level of satisfaction with the relationship with your family in the last 12 months?	PAECO - In the last 12 months have you attended meetings of environmental or civil rights association?
	PCULT - In the last 12 months have you attended meetings of cultural or recreational association?

Following the related literature, as not only social capital impacts health and healthcare utilization, we then selected some demographic variables that represent important predictors of our dependent variables, such as age, gender, location and education. We included self-management variables by considering the two that were available (salt and weight control frequency), and health status variables (presenting chronic disease status). We also have built a variable to indicate physical activity, based on the respondent’s answers about performing domestic/labor physical activity or practicing sport.

Regions of residence were grouped in North, Centre and South. Due to data privacy restrictions, we did not have access to the exact municipality of each participant, only to the regional level; and our analysis was aggregated likewise.

Finally, we selected two outcome variables. First, the self-reported health status, represented by a Likert scale question asking the respondents to assess their health in general by the time of the interview. This variable takes values from one to five, from “very bad” to “very good”. The second outcome is the healthcare utilization, evaluated in two additional levels: the first as the number of unplanned care accesses here representing the sum of the answers of whether the respondent has used emergency care (*pronto soccorso*) or after-hours medical care and services (*guardia medica*); and the second represented by the count of overnight hospital stays (both planned or unplanned), all answered as counts in the last three months. Sample’s summary statistics are presented in Table 1 on the Supplementary Material.

### Model and statistical analysis

**Fig. 1:**
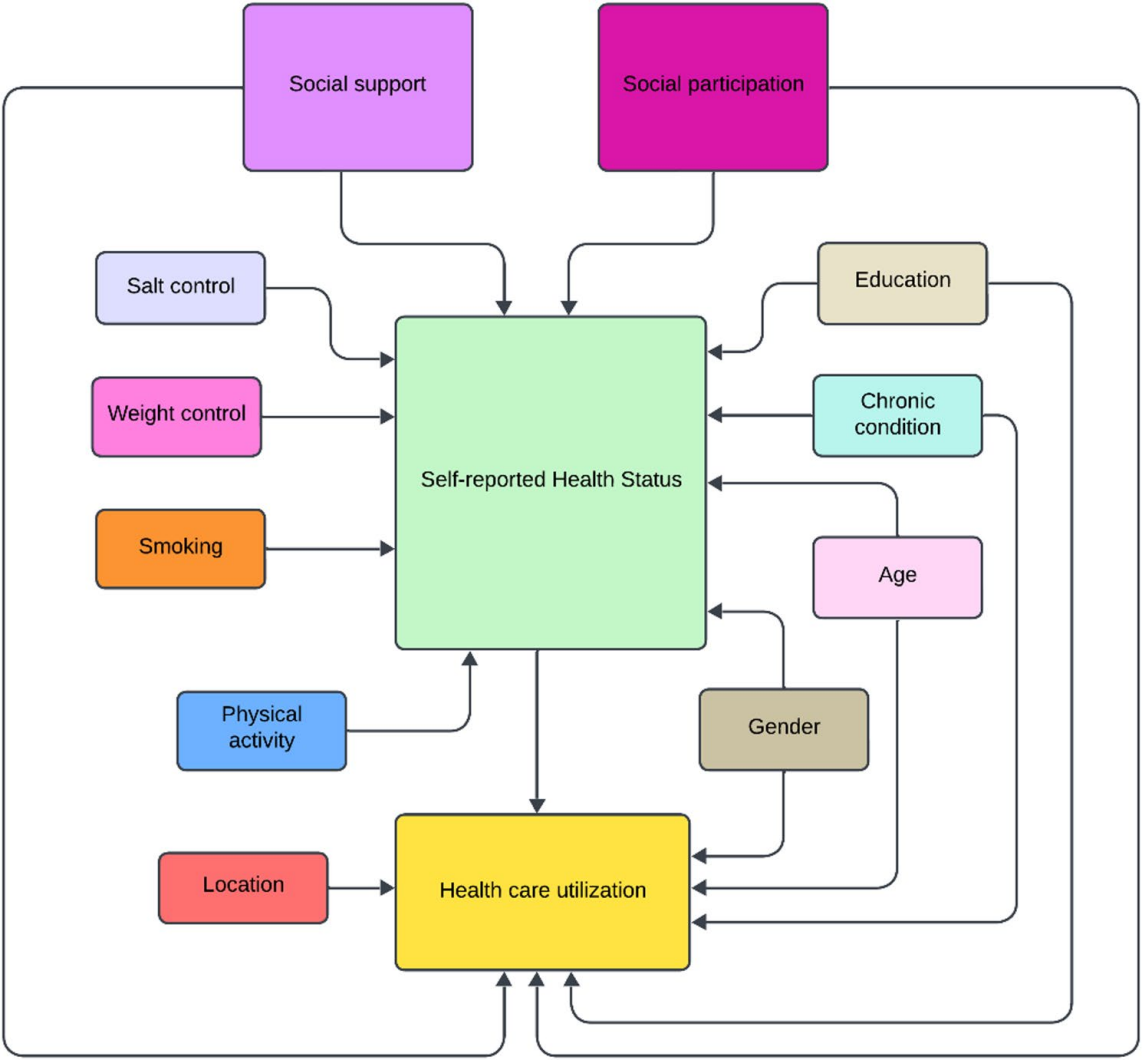
Model of structural equations

For the mediation analysis, the framework of Structural Equations Model (SEM) was adopted. SEM are models that embrace statistical techniques and the theoretical relationship behind the data, where the path diagrams correspond to systems of regressions, indicating the expected relationships between the variables. On SEM models, each equation from the system is estimated using a linear regression, and the whole system can represent endogenous and chained connections by selecting several exogenous variables mixed with variables determined within the model, based on a theoretical rationale and identification of the system [32, 33]. However, it is worth saying that our cross-sectional design indicates relationships that suggest, although cannot guarantee causality. Our proposed path diagram is represented in Fig. 1 below.

Following the theoretical explanations on the possible connections of social capital and health, our hypothesis is that social capital may enhance well-being directly, while it could also affect health care utilization directly and indirectly via health status. We consider the direct and indirect effects of the remaining sociodemographic variables to account for possible observable features that might influence this relationship as potential confounding factors. For identification reasons, we could not use all the same control variables in each equation, this is the reason why we prioritized the relationships aligned to our main research hypothesis and relevant literature discussed in the last section.

Self-management variables (salt control and weight control) were included to represent proxies of self-awareness and self-control, which may impact one’s health. Besides, empirical literature has already pointed out that social capital may influence health through behaviors [10, 14, 16, 20]. As they might be important confounding factors, we are choosing to control for them as to better identify our main hypothesis and focus on health status and health care utilization. In addition, we also included smoking status as a representation of behavioral elements that contribute to changes on the perceived health status and then on health care utilization.

Age, education and gender were included to represent sociodemographic influences in the model. These variables entered on the two equations because they are assumed to influence both health and access to health care. Instead, location was modelled to relate to health utilization alone because we assume it is intrinsically linked to health care access. Additionally, the inclusion of location in the model is justified by the relevant literature claiming the outstanding heterogeneity between the Italian regions in terms of access and quality of care [1–3].

Absence or prevalence of chronic diseases and multimorbidity are important predictors of both health status and hospital visits, and this is the reason why we included them on both equations. Not only that, but we have a special interest in understanding whether they play a most significant role in the first or in the second equation.

The description of both equations following the path diagram is presented below. All statistical analysis were performed using R package “lavaan” [34].

1 $$\begin{aligned} HStatus & =\alpha \cdot Socia{l}_{support}+\beta \cdot Socia{l}_{participation} \\ &\quad +Salt+Activity+ Weight+Smoking\\ &\quad +Age+Gender+Educ+ Chronic \end{aligned}$$

2 $$\begin{aligned} UnplanHosp & =\gamma \cdot Socia{l}_{support}+\delta \cdot Socia{l}_{participation} \\ & \quad +Location+Age+Gender+Educ \\ & \quad +Chronic+\rho \cdot HStatus \end{aligned}$$

Where the estimated parameters allow us to identify the respective mediating effects. The multiplication $\alpha \cdot \rho$ represent the indirect effect of social support via health status and $\beta \cdot \rho$ represent the indirect effect of social participation via health status. In their turn, $\alpha\cdot \rho+\gamma$ represent the total effect of social support and $\beta\cdot \rho +\delta$ accounts for the total effect of social participation.

## Results

Table 2 displays the results of the two equations system for healthcare utilization. The fitting statistics are within the thresholds for SEM estimates [35–36], indicating that the system of equations converged, and the model fitted well to the data. We chose the DWLS estimator (Diagonal weighted least squares) because it is more robust to non-normality and the most appropriate to categorical/ordinal variables in the outcome [37].

**Table 2:** SEM results

Equation’s system	Covariates	Unplanned care visits (A)	Overnight hospital stays (B)
		Estimate (sd)	Estimate (sd)
First equation - Perceived health status			
	Social support	0.125*** (0.001)	0.125*** (0.001)
	Social participation	0.024*** (0.002)	0.024*** (0.002)
	Salt control	0.002* (0.002)	0.002* (0.001)
	Weight control	0.011*** (0.001)	0.011*** (0.001)
	Physical activity	0.087*** (0.002)	0.088*** (0.002)
	Smoking status	−0.016*** (0.001)	−0.016*** (0.001)
	Age	−0.068*** (0.001)	−0.068*** (0.001)
	Gender	−0.071*** (0.002)	−0.071*** (0.002)
	Education	0.056*** (0.001)	0.056*** (0.001)
	Chronic condition	−0.438*** (0.002)	−0.438*** (0.002)
Second equation – Health care utilization			
	Perceived Health Status	−0.256*** (0.004)	−0.323*** (0.006)
	Social support	0.008*** (0.004)	0.034*** (0.005)
	Social participation	0.034*** (0.006)	−0.024*** (0.008)
	Region - North	0.055*** (0.008)	0.037*** (0.012)
	Region - South	−0.023* (0.009)	−0.021* (0.013)
	Age	−0.033*** (0.002)	0.015*** (0.003)
	Gender	−0.024*** (0.006)	−0.057** (0.011)
	Education	−0.061*** (0.004)	−0.021*** (0.005)
	Chronic condition	0.148*** (0.005)	0.136*** (0.008)

Fitting statistics			
Estimator	DWLS		DWLS
Number of model parameters	26		25
Number of observations	336,592		336,461
Number of iterations to convergence	73		65
Degrees of freedom	6		6
CFI	0.954		0.915
TLI	0.992		0.986
RMSEA	0.009		0.012
SRMR	0.000		0.000

Standard errors in parentheses *** *p* < 0.01, ** *p* < 0.05, * *p* < 0.1. DWLS – Diagonal weighted least squares. CFI – Comparative fit index. TLI – Tucker-Lewis index. RMSEA - Root Mean Square Error of Approximation. SRMR - Standardized Root Mean Square Residual

For the first equation both levels of healthcare utilization hold the same results. Both dimensions of social capital - social support and social participation - were found significantly related to perceived health status, but social support held a bigger importance. Weight control and physical activity were significant and positively related to reported health status, while salt control was only significant at the 10% level. These results show that behavioral variables are linked to better perceived health status.

Education’s coefficient was positive, indicating a positive relationship between education and health status. Smoking and age are, as expected, negatively related to better health status, both statistically significant at the 1% level. The biggest impact on perceived health status is the presence of chronic illnesses: the coefficient is highly significant. This indicates that a considerable impact on perceived health status comes from established conditions related to the presence of chronicity.

The second equation presents healthcare utilization variables. Model (A) shows the unplanned care accesses as dependent variable while Model (B) displays the results for overnight hospital stays. The variables that influenced health status on the first equation could affect healthcare utilization and could also be mediated through the perceived health status. Perceived health status itself is the main predictor for both models, being significantly related to unplanned care accesses and overnight hospital stays, showing that better health is connected to less health system’s usage. This coefficient also reinforces the pathway that theoretically links the first to the second equation. Chronic condition variable is positively related to more visits in both models, as expected. This means that chronic patients display a higher likelihood of using health care services even after controlled for their own health status. From the magnitude of these coefficients, we can attest that the biggest impact of chronicity comes from the loss of perceived health instead of the frequency of healthcare visits.

We found that social support, besides improving health, presents a positive relationship with healthcare utilization. We hypothesize these effects are connected with some sort of pushing effect from family and friends towards the seek for care once someone has an unplanned care access or an overnight hospital stay need; also, this may be interpreted as a residual effect once the influence from the established disease and health status was ruled out thanks to the inclusion of the remaining covariates in the second equation.

Interestingly, social participation displays different coefficients for our two levels of health utilization variables. It was found that meanwhile social participation is positively related to health utilization (emergency and out-of-hours services), it is negatively related to the number of overnight hospital stays. We theorize that having a strong network - resulting in a bigger social participation score - can benefit those who have emergent needs in accessing these services via pushing effect, while the preventive effect might dominate those who seek overnight hospital services.

North Italy compared to both Center and South, showed a significant increase in healthcare utilization, potentially due to the higher services supply. Gender continued to present a negative relationship with the outcome, indicating that after controlling for the health status effects, males tend to make greater use of the health care system. Education was found to have a negative effect on both access categories. Interesting results show that while age displays a negative coefficient for the unplanned care visits it has a positive coefficient for overnight hospital stays, probably due to age-specific needs.

Further tests using total health utilization were done and pointed out in the same direction as unplanned care access. The results are depicted in Table S.3 in the Supplementary material.

Table 3 presents the estimates of indirect and total effects derived from the parameters from the linear regressions of Eqs. (1) and (2). We found that the indirect effects of social capital variables are all negative, thus meaning that both dimensions can affect health utilization via health status indirectly, decreasing its use with statistical significance.

Considering this indirect effect and the direct effects altogether, the total effects point to social support decreasing unplanned care accesses, but the net effects for overnight hospital stays are non-significant. Hence, once we take into consideration the care seeking behavior pushed from social support, the effects are cancelled out. Instead, the net effect of social participation is positive for unplanned care access and negative for overnight hospital stays; thus, meaning that social participation’s pushing behavior is dominant for unplanned care access and the protective effect is dominant for overnight hospital stays.

**Table 3:** Indirect and total effects of social support and social participation in health status and health utilization

Unplanned care access		
	Indirect effect via health status	Total effects (direct + indirect)
Social support	−0.032*** (0.001)	−0.024*** (0.004)
Social participation	−0.006*** (0.00)	0.031*** (0.005)

Overnight Hospital stays		
	Indirect effect via health status	Total effects (direct + indirect)
Social support	−0.040*** (0.001)	−0.006 (0.005)
Social participation	−0.008*** (0.01)	0.032*** (0.008)

Parameters tested using Wald test. Standard errors in parentheses *** *p* < 0.01, ** *p* < 0.05, * *p* < 0.1

## Discussion

In this work, we proposed item-response methods to estimate two multidimensional social capital variables. Following the related social capital theories, we choose to focus on social support and social participation. Our results indicate that such life dimensions play an important role in improving health outcomes, proxied by perceived health status.

However, when it comes to translating these benefits into a decrease of health services usage, there is a compensating influence that stems from the pushing effect towards care seeking behavior, and the results are mixed – they depend on the type of social capital and type of care.

The disentanglement of these effects is crucial for two reasons: firstly, one should not take for granted that social capital has only one single impact on health care utilization via benefits on health care status. Indeed, the effects towards care seeking are strong and dominate some of the effects we have found. Secondly, the interrelation between these variables and the remaining variables of our SEM indicates that this relationship is still present even after controlling for important possible confounding factors.

The channels that may explain these findings are supported by theories of social capital as a tool for improving help seeking and health literacy, which agrees with previous reviews and empirical findings [13, 14, 17, 19]. At the same time, the hypothesis pointing to well-being benefits from social capital are also part of the explanation and are related to the findings of the effect on self-perceived health status. These results are also in line with the relevant literature [7, 9, 13, 16, 24–26]. Our results may indicate that both approaches have empirical sounding.

Regarding the remaining covariates, education was a very important predictor in both equations. As already mentioned in previous literature, higher education is connected to higher perceived health status [10, 38]. The novelty of our paper was to find that even after considering the effects on health status, education is also a significant factor in decreasing care accesses. Our results go in the same direction as previous findings [39]. We believe that the residual protective benefits of education overcome the direct effect from improvements on health status alone.

As in other studies [38], also here males report a better perceived health. Some studies [40] did not find differences in the gender utilization of health care services and others found males use it more [41]. In our study, males displayed a slightly bigger health utilization after controlling for the health status in the second equation.

The results for age were as expected for the first equation, indicating that older age respondents are likely to report a lower score of health status. However, in the second equation, the results diverge. On one hand, younger people experience more unplanned care accesses, on the other hand, older people are predominant amongst those who had hospital overnight stays. Although other studies have found before that older individuals present a higher utilization of emergency services [42], these results were only visible for our outcome of overnight hospital visits. This may derive from the fact that the first equation had controlled most of the effects of age on health status; henceforth the second equation can represent primarily the non-age-health-related unplanned care accesses. For the overnight hospital stays instead, the age effect predominates. One reason that support this explanation is the fact that in Italy the emergency department visits are dominated by adults up until 49 years old [40] while overnight hospital stays are largely represented by elderly [43].

Self-management variables and physical activity were, as expected, correlated with health status, agreeing with other findings [10, 44–46]. Smoking status, also, presented the expected direction of results in line with the literature [47, 48].

An important finding relates to chronicity. There is a sounding literature linking health status and chronicity or health utilization and chronicity inside [49]– [50] and outside Italy [12, 29]. In our study, chronic and multimorbid patients had a significant decrease in their perceived health status, and even after controlling the health status effects, they frequent healthcare services more. Although we acknowledge that there may exist a gap between the health status in the moment of the health service utilization and the moment when the survey took place, this finding shows a relevant link between chronicity and health care utilization not only for the patients with poorer health status; but also, for all the ones affected by established diseases despite how they might feel.

Italian regions have their own decentralized health systems [1, 2], thus it was justifiable including the location as a relevant factor on our equation’s system. The Italian universal health system is designed to guarantee equitable access to care across all regions, yet persistent territorial and social inequalities continue to undermine this goal [51]. Recent studies point out that residents in southern Italy remain markedly less likely to receive specialist visits or diagnostic tests than those in the central or northern regions, even when they possess adequate economic resources [51]. Our results agreed with such findings, as the frequency of care accesses was higher in the North compared to the Center and South. Among the reasons that may explain this phenomenon there is the concentration of resources in the northern regions [1], heterogeneity of access and quality and cultural differences in health-seeking behavior. Moreover, unobserved region-specific factors, such as administrative efficiency, local governance, and historical patterns of investment—further contribute to uneven health outcomes. We hope that future research will explore in greater depth the complex interrelations between social capital, health status, and health care utilization from the perspective of regional and sub-regional inequality.

This research was conducted using Italian data, with a nationally representative sample derived from ISTAT repeated survey. Although such configuration limits direct generalizability with other countries, other European surveys ask similar questions that could provide some level of comparability. This is the case of European Social Survey, which cover around 30 countries, and the Survey of Health, Ageing and Retirement in Europe (SHARE), covering 27 countries. We hope our methodological approach of using IRT to define social capital indexes may inspire future works in other settings.

Still, our research has some limitations. Firstly, disaggregated information by municipality or province was not available, limiting more detailed analysis. Lack of data availability regarding the reason for these hospital visits, and its consequences for the individual, has restricted a detailed analysis. Such a limitation implies two relevant caveats: for the hospital stays, it means it is impossible to disentangle elective visits from the others. Elective visits may represent treatment for established conditions, which can have a positive impact on the self-rated health status. In contrast, when it comes to emergency department visits, we should recognize that more frequent use does not necessarily imply that these visits were truly needed. As previous studies have shown, inappropriate use of emergency services can be a significant issue in Italy [52]. Further research might focus on this separation by additional information such as ICD9, type of hospitalization, and triage code, following the patient’s trajectory within the health system, and trying to explore more deeply the drivers of the pushing or preventive behavior into care seeking.

The data sources used a sampling strategy of cross-sectional survey, which invalidated the possibility of using longitudinal analysis as individuals were not followed through time. A phenomenon we could not reject is the possibility of social capital and health presenting a bi-causal relationship, but, again, such hypothesis is untestable with the available cross-sectional data. Furthermore, we acknowledge that there may be several other relevant variables (i.e., personal income, distance from the hospital, etc.) who would be expected to interact with our effects, but lack of available data had limited exploring this further. Finally, we do not exclude the possibility of recall and recency bias being present in the respondents’ answers due to the design of self-reported responses.

Social capital theories accounting links with health generally focus on social support as an example of informal social capital; and social support is already an established determinant of health. However, formal social capital can be expressed in many ways: social networks, trust, political participation among others. Following the work from Durante et al. [22], we selected social participation in the construction of our measure because the authors have already identified as potentially correlated with health. Nevertheless, we acknowledge that other components of social capital may interact with health outcomes. We did a few tests using the other modules from Durante et al. [22], and although some of the results were promising, statistical significance and theory-sounding were more robust for the two we chose. We hope future works may expand the role of other social capital components in improving health outcomes in Italy.

It is also important pointing out that post-COVID years may have impacted health behaviors permanently. We hope these possible behavioral changes can be absorbed by our observed covariates, but we acknowledge these effects might not be completely ruled out. We also acknowledge that COVID-19 have changed the patterns of healthcare utilization in Italy, as well pointed out in the literature [40]. Due to the design of the SEM estimation, fixed effects by year would bring difficulty in reaching the identifiability and convergence of the system, thus we preferred not to include it. Although the affected years should be only 2020 and 2021, our results may present some downward bias and should be interpreted as the lower bound estimates.

## Conclusion

Social support and social participation are two representations of social capital. In this study, we found that such measures can impact health outcomes and health utilization in Italy, both directly and indirectly.

These findings provide relevant policy implications. The need for social connections is inherent to human beings, and there are outstanding benefits for the self-perceived health status. This evidence supports policies that identify individuals’ social needs and encourage the government to actively back third-sector initiatives aimed at bringing people together, foster social connections, encourage participation in community activities, build intergenerational community cohesion.

Though, there are other effects driven by care seeking behavior that should be considered. Especially, it is important to promote good quality health information and improve health literacy so that the support net can be effective in providing accurate information and help when needed. On the other hand, individuals with low quality and quantity of social capital are at risk of both having a lower health status and not accessing the healthcare system due to this lack of support. Especial attention should be given to this population to ensure and enhance equality in Italy.

## Supplementary Information

Supplementary Material 1

## Data Availability

All data is publicly available in the cited sources. For specific requests, please contact the main author or the corresponding author.

## Footnotes

1 The multiscope survey started in 2008, but many of the variables we were particularly interested in were available only from 2014 on.
