# Supplementary material for: Mediating effects between social capital and health care utilization in Italy–a structural equation model analysis
Source: Popul Health Metr. 2025 Dec 20;23:75. doi: 10.1186/s12963-025-00441-6 (PMC12750791; doi:10.1186/s12963-025-00441-6)
Supplement: Supplementary file 1 — Supplementary Material 1 [file 12963_2025_441_MOESM1_ESM.docx]

## Supplementary Material

IRT information

Figure S.1 – IRT Characteristic curves for social support


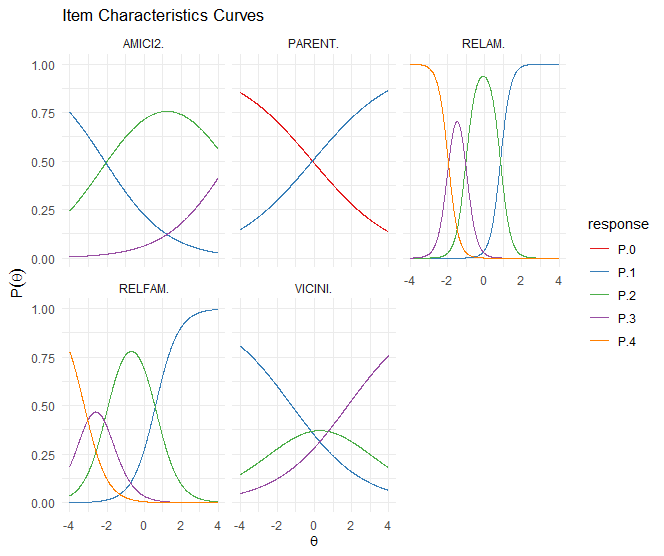
Parameter theta ($\theta$) represent the latent trait “Social support”. The parameter discrimination ($\beta$) and difficulty ($\delta$), evaluated from the curves, show that the answers of the respondents display a marked difference allowing for the index to represent the latent trait. Loadings and values of the parameters available in Table S.1.

| Fitting statistics: |  |
| --- | --- |
| Iterations to convergence | 117 |
| Method | Single factor |
| Log-likelihood | -1208786 |
| Estimated parameters | 16 |
| AIC | 2417604 |
| BIC | 2417772 |
| RMSEA | 0.039 |

Figure S.2 – IRT Characteristic curves for social participation
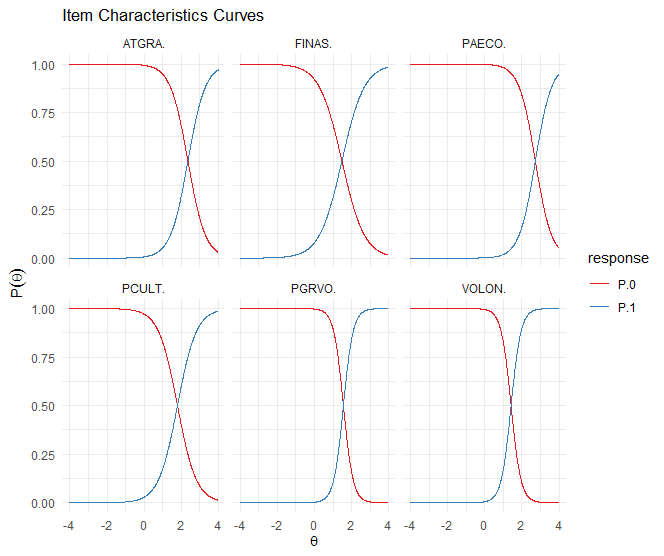


Parameter theta ($\theta$) represent the latent trait “Social Participation”. The parameter discrimination ($\beta$) and difficulty ($\delta$), evaluated from the curves, show that the answers of the respondents display a marked difference allowing for the index to represent the latent trait. Loadings and values of the parameters available in Table S.1.

| Fitting statistics: |  |
| --- | --- |
| Iterations to convergence | 21 |
| Method | Single factor |
| Log-likelihood | -380013.1 |
| Estimated parameters | 12 |
| AIC | 760050 |
| BIC | 760176 |
| RMSEA | 0.055 |

Table S.1 – Parameters from the IRT

| Social support | Parameter a (loadings) | Parameter b (discrimination by item) |
| --- | --- | --- |
| AMICI2 | 0.587 | -2.124 / 4.6 |
| PARENT | 0.451 | -0.107 |
| VICINI | 0.516 | -1.224 / 1.798 |
| RELAM^£^ | -3.670 | 0.844 / -1.020 / -1.973 |
| RELFAM^£^ | -1.612 | 0.619 / -1.971 / -3.229 |
| Social participation |  |  |
| FINAS | 1.648 | 1.479 |
| VOLON | 3.727 | 1.414 |
| ATGRA | 2.119 | 2.366 |
| PGRVO | 3.805 | 1.563 |
| PAECO | 2.284 | 2.723 |
| PCULT | 1.959 | 1.802 |

^£ -^ Both RELFAM and RELAM are measured in decrescent manner, thus justifying the negative loadings in the composite model.

Figure S.3 – Item’s correlation map

1. Social support


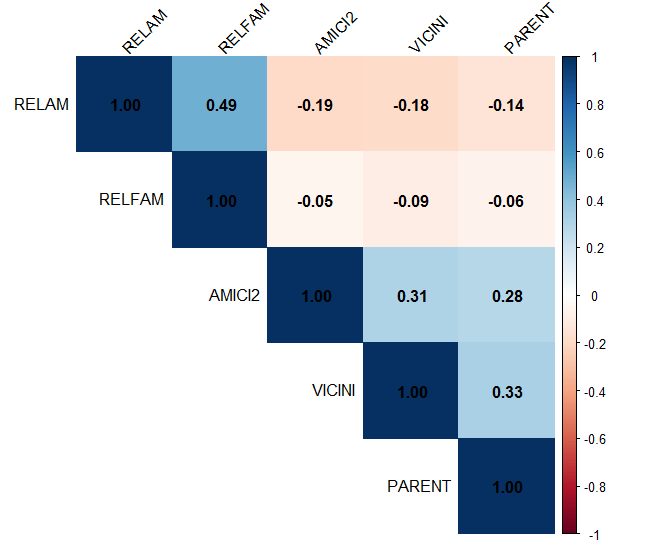


Our correlations’ matrix for the items that compose social support indicates that variables about the quality of relationships correlate moderately between them, while variables about the quantity of relationships correlate moderately between them. Quality and quantity of relationships, instead, present a very low correlation. This pattern indicates they are two different dimensions of social support, thus supporting our decision to include both types quality and quantity variables in our composite index.

1. Social participation


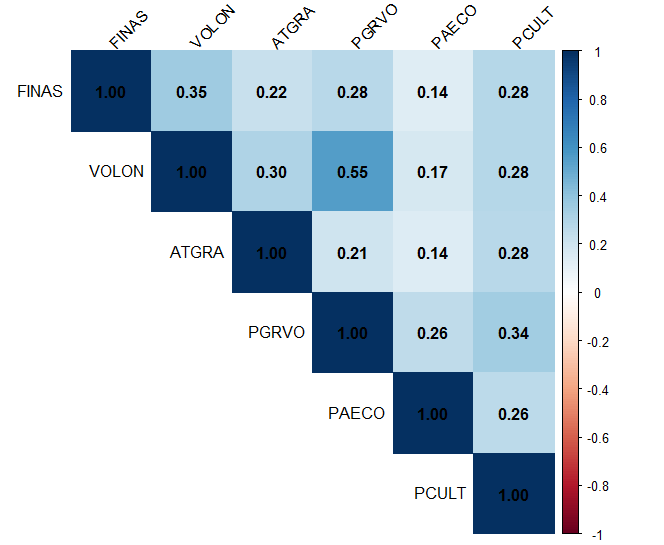


Our correlations’ matrix for the for the items that compose social participation present mostly moderate correlations. Thus, we have evidence that they may compose a latent trait of social participation together.

1. IRT social participation x IRT social support


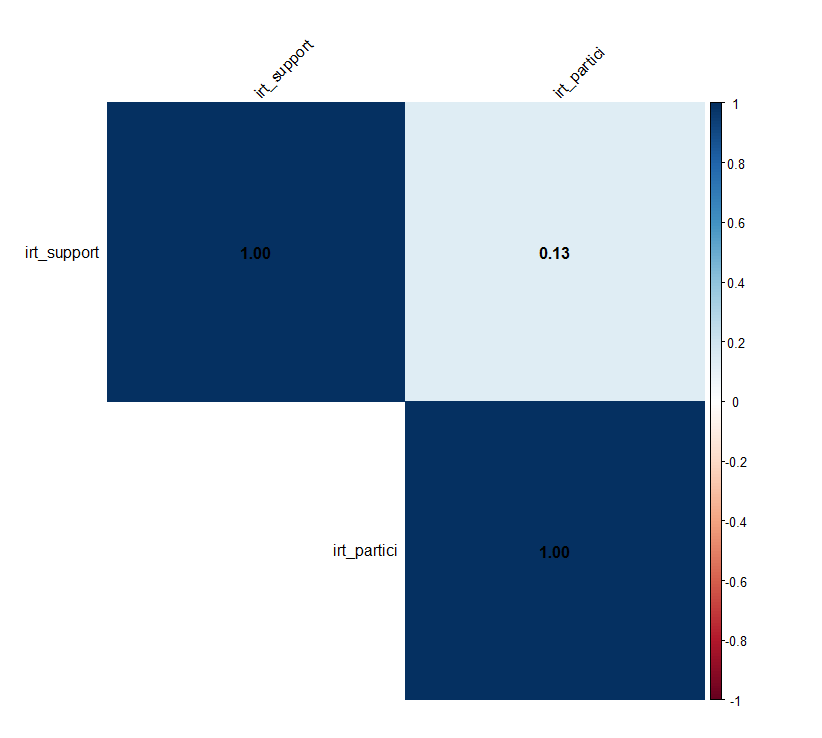


The low correlation between the indexes indicates they probably do not overlap and/or represent the same latent traits, henceforth supporting the division of social capital into these two measures instead of one.

Table S.2 – Summary statistics

| Variable | N = 354,256 |
| --- | --- |
| Age in years |  |
| From 18 to 24 | 27,742 (7.8%) |
| 25 to 34 | 40,450 (11.4%) |
| 35 to 44 | 53,795 (15.2%) |
| 45 to 54 | 66,679 (18.8%) |
| 55 to 59 | 32,017 (9%) |
| 60 to 64 | 29,044 (8.2%) |
| 65 to 74 | 52,292 (14.8%) |
| 75 and over | 52,237 (14.7%) |
| Gender |  |
| Male | 168,725 (47.6%) |
| Female | 185,531 (52.3%) |
| Education level |  |
| No formal education, elementary school diploma | 62,315 (17.6%) |
| Middle school diploma | 99,387 (28.1%) |
| High school diploma | 136,373 (38.6%) |
| University degree and postgraduate degree | 55,081 (15.7%) |
| Region |  |
| North | 150,772 (42.5%) |
| Center | 80,313 (22.7%) |
| South | 123,171 (34.8%) |
| Smoking status |  |
| No smoker | 197,034 (55.7%) |
| Ex smoker | 88,069 (24.9%) |
| Currently smoker | 68,516 (19.4%) |
| Regulates salt intake in your diet |  |
| No | 85,100 (24.2%) |
| Yes, I have been decreasing | 136,996 (38.9%) |
| Yes, I have always paid attention | 129,621 (36.9%) |
| How frequently do you weigh yourself? |  |
| Never | 63,736 (18.4%) |
| A few times per year | 109,056 (31.5%) |
| Once a month | 90,262 (26%) |
| Once a week | 66,768 (19.3%) |
| Everyday | 16,672 (4.8%) |
| Physical activity^£^ |  |
| Yes | 158,347 (45%) |
| No | 195,909 (55%) |
| Chronic condition^¥^ |  |
| No | 188,838 (53.3%) |
| Yes, one | 115,633 (32.6%) |
| Yes, more than one | 49,785 (14.1%) |
| How is your health going in general? |  |
| Bad  or Very bad | 24,176 (6.8%) |
| Neither bad nor good | 104,393 (29.5%) |
| Good | 175,488 (49.5%) |
| Very Good | 50,199 (14.2%) |
| Number of unplanned care accesses |  |
| 0 | 319,268 (91%) |
| 1 | 25,745 (7.3%) |
| 2 | 3,633 (1%) |
| 3 or more | 1,586 (0.4%) |
| Number of overnight hospital stays |  |
| 0 | 340,174 (97.2%) |
| 1 | 8774 (2.5%) |
| 2 | 796 (0.2%) |
| 3 or more | 323 (<0.1%) |
| Social support index |  |
| mean | 0 |
| max | 1.71 |
| min | -2.52 |
| Social participation index |  |
| mean | 0 |
| max | 2.71 |
| min | -0.36 |

£ - Comprising the occurrence of at least 180 minutes of physical activity based on the answers of domestic physical activity, labor physical activity and practicing sport regularly.

¥ - Comprising the following chronic diseases: Diabetes, Hypertension, Infarct, Angina or other cardiologic diseases, Bronchitis, Asthma, Tumor, Ulcer, Cirrhosis, Arthrosis, Osteoporosis, Parkinson, Alzheimer and other neurological disorders
